# Incidence of concomitant chondral/osteochondral lesions in acute ankle fractures and their effect on clinical outcome: a systematic review and meta-analysis

**DOI:** 10.1007/s00402-020-03647-5

**Published:** 2020-10-31

**Authors:** Ali Darwich, Julia Adam, Franz-Joseph Dally, Svetlana Hetjens, Ahmed Jawhar

**Affiliations:** 1 Department of Orthopaedics and Traumatology Surgery, University Medical Centre, Medical Faculty Mannheim of the University of Heidelberg, Theodor-Kutzer-Ufer 1-3, 68167 Mannheim, Germany; 2 Institute of Medical Statistics and Biomathematics, University Medical Centre, Medical Faculty Mannheim of the University of Heidelberg, Mannheim, Germany; 3 Department of Trauma, Hand and Reconstructive Surgery, Klinikum Worms, Academic Teaching Hospital of the University Mainz, Worms, Germany

**Keywords:** Ankle fracture, Chondral/osteochondral lesions, Cartilage lesion, Incidence, FAOS, AOFAS

## Abstract

**Introduction:**

Despite successful osteosynthesis, some patients report residual symptoms after ankle fractures. One of the reasons behind the postoperative complaints might be traumatic concomitant chondral lesions (CL) and/or osteochondral lesions (OCL) within the ankle joint. The study aims to systematically review the incidence of CL and/or OCL in ankle fractures and to assess their effect on the clinical outcome.

**Materials and methods:**

This work was conducted according to PRISMA checklists. A systematic literature search was performed using following keywords: “Ankle Fractures” OR “Trimalleolar Fracture” OR “Bimalleolar Fracture” OR “Maisonneuve fracture” OR “Malleolus Fracture” AND “Cartilage” OR “Cartilage Diseases” OR “Cartilage, Articular” OR “chondral” up to March 2020. The identified articles were analysed to determine the incidence of CL and/or OCL. Included studies in the meta-analysis assessed possible cartilage damage through arthroscopy or MRI immediately after traumatic ankle fractures and described the postoperative clinical outcome.

**Results:**

The search identified a total of 111 publications; 19 described the incidence of CL and/or OCL after ankle fractures; six met the criteria to be included in the meta-analysis: five (*n* = 293) diagnosed CL and/or OCL through arthroscopy during ORIF and one study (*n* = 153) used preoperative MRI. The clinical outcome was evaluated in four studies (*n* = 177) using AOFAS score and in two (*n* = 269) using FAOS score. The mean incidence of arthroscopically detected CL and/or OCL was 65 ± 21% [95% CI 53.9 to 76.72]. The cumulative meta-analysis sample size comprised a total of 400 Patients (170 with and 230 without CL and/or OCL) available for a mean follow-up of 23.9 ± 11.5 months [95% CI 11.79 to 36.07]. The average age was 44.3 ± 5.5 years [95% CI 38.57 to 50.13]. The meta-analysis revealed a mean AOFAS score of 91.2 ± 4.8 [95% CI 83.53 to 98.93] with versus 94.4 ± 4.7 [95% CI 86.81 to 102.07] without CL and/or OCL (*p* = 0.15) and a mean FAOS score of 73.2 ± 11.31 [95% CI − 28.44 to 174.85] with versus 79.0 ± 18.4 [95% CI − 86.77 to 244.87] without CL and/or OCL (*p* = 0.18).

**Conclusions:**

CL and/or OCL appear very frequently after ankle fractures. A tendency towards a favourable short- to mid-term clinical outcome was noticed in ankle fractures without CL and/or OCL, however without reaching statistical significance.

**Level of evidence:**

Level I.

## Introduction

Ankle fractures are one of the most common injuries of the lower limb and have a yearly incidence of 0.1–0.2% [1–4]. It has been frequently shown that good to excellent results are reached, when treating unstable ankle fractures with open reduction and internal fixation (ORIF) [2, 5]. Anatomical reduction, stable internal fixation and restoration of the ankle stability are the main objectives of the operative treatment. Despite achieving these objectives, numerous patients continue to report residual symptoms such as recurrent swelling, persistent pain or compromised range of motion [6–8]. These residual complaints may be due to concomitant chondral lesions (CL) and/or osteochondral lesions (OCL) occurring during the initial trauma. Trauma has been repeatedly shown to be the leading cause of CL and/or OCL [3, 4, 9], however, the incidence of CL and/or OCL with ankle fractures is roughly estimated and has been reported to vary widely between 17% and as high as 89% [6, 10, 11].

These lesions are thought to be frequently missed immediately after trauma or delayed diagnosed, which may lead to joint degeneration and chronic pain. 14–50% of patients with CL and/or OCL develop posttraumatic osteoarthritis [12–18]. In comparison with other joints of the lower limb, the incidence of posttraumatic osteoarthritis of the ankle is the highest. In the work of Saltzman et al. [19], 639 patients with symptomatic arthritis of the hip, knee or ankle (Kellgren–Lawrence grade 3 or 4 [20]) presenting in a time period of 1 year were analysed. Aetiology of the arthritis was determined through medical history and physical examination. 54% of the patients presenting with ankle osteoarthritis reported history of trauma (including fractures, sprains with continued pain and recurrent sprains with instability as well as osteochondrosis dissecans) compared to 8% in the hip and 12.5% in the knee.

Some studies evaluated the role of arthroscopy [13, 14, 18, 21–27] and of imaging diagnostics (MRI; CT; Arthrography) [12, 28, 29] in the assessment of cartilage damage. Other studies focused on the evaluation of patients` outcome after ankle fractures regardless of the extent of cartilage damage [21, 25, 30]. Few authors assessed the incidence of CL and/or OCL after traumatic ankle fractures and their clinical outcome [1, 12, 31–34].

Thus, the clinical significance of CL and/or OCL after ankle fractures remained unclear. The present study is the first to determine systematically the incidence and the clinical significance of CL and/or OCL after ORIF of ankle fractures.

The hypothesis of the meta-analysis was that CL and/or OCL after ankle fractures negatively affect the postoperative outcome.

## Review

### Materials and methods

This work was conducted according to the PRISMA (Preferred Reporting Items for Systematic Reviews and Meta-Analyses) checklists and guidelines [35].

### Search strategy

A systematic literature search strategy was applied to the following databases: PubMed, Ovid Medline, the Cochrane Library, Web of Science and PsycInfo (via EBSCO). The PICO Model was used while performing the search [36]. Used keywords included:

“Ankle Fractures” OR “Ankle Fracture” OR “Trimalleolar Fracture” OR “Bimalleolar Fracture” OR “Maisonneuve fracture” OR “Malleolus Fracture”

AND

“Cartilage” OR “Cartilage Diseases” OR “Cartilage, Articular” OR “cartilage” OR

“chondral”.

The search was performed by a qualified medical librarian and revised/completed on 01.03.2020.

### Study selection and eligibility criteria

The 98 identified studies were screened by three of the authors (AD, JA and AJ) through reviewing title and abstract of each study. Relevant articles were then included after reading the full text and identifying the required parameters. Furthermore, reference lists of the selected studies were inspected for additional relevant articles. An additional 13 publications were identified in this context.

There were no language limitations in the selection of the articles. Exclusion criteria involved: paediatric patients under 18 years (one study), case reports (three studies), surgical techniques and/or overviews of treatment options (20 studies) and experimental studies on animals (one study) or on cadavers (four studies). 17 Studies, that assessed cartilage damage at a postoperative stage or included cartilage damage of degenerative origin, were excluded. Studies not recording cartilage damage (30 studies) or without clinical follow-up were excluded (12 studies). Four studies [14, 18, 23, 30] used different outcome measures and were, therefore, not eligible to be included in the meta-analysis. Lantz et al. [30] used a 100-point system, Thordarson et al. [18] used the SF-36 and MODEMS score, Ono et al. [14] rated his patients according to the method of Burwell and Charnley and Fuchs et al. [23] used the Olerud and Molander ankle fracture scoring system.

### Endpoints

Assessment of cartilage damage was done through arthroscopy as part of the operation (ORIF) or through MRI immediately after trauma/preoperatively.

All studies were screened by three of the authors (AD, JA and AJ) to determine the incidence of CL and/or OCL in the setting of ankle fracture. Based on these findings, a weighted mean value and range for the incidence of CL and/or OCL was calculated.

Included studies in the meta-analysis evaluated the clinical outcomes using FAOS or AOFAS.

FAOS is a valid and reliable score [37] that ranges from 0 (severe symptoms) to 100 (no symptoms) and comprise five subscales for the patient´s subjective self-assessment: Pain, Symptoms, function in activities of daily living (ADL), function in sports and recreation (Sports), and overall foot-and-ankle-related quality of life (QoL).

AOFAS is a well-established and commonly used score [38] including subjective as well as objective clinical parameters and ranging from 0 (severe symptoms) to 100 (no symptoms). It comprises three major categories: pain, function and alignment.

### Statistical analysis

**Fig. 1:**
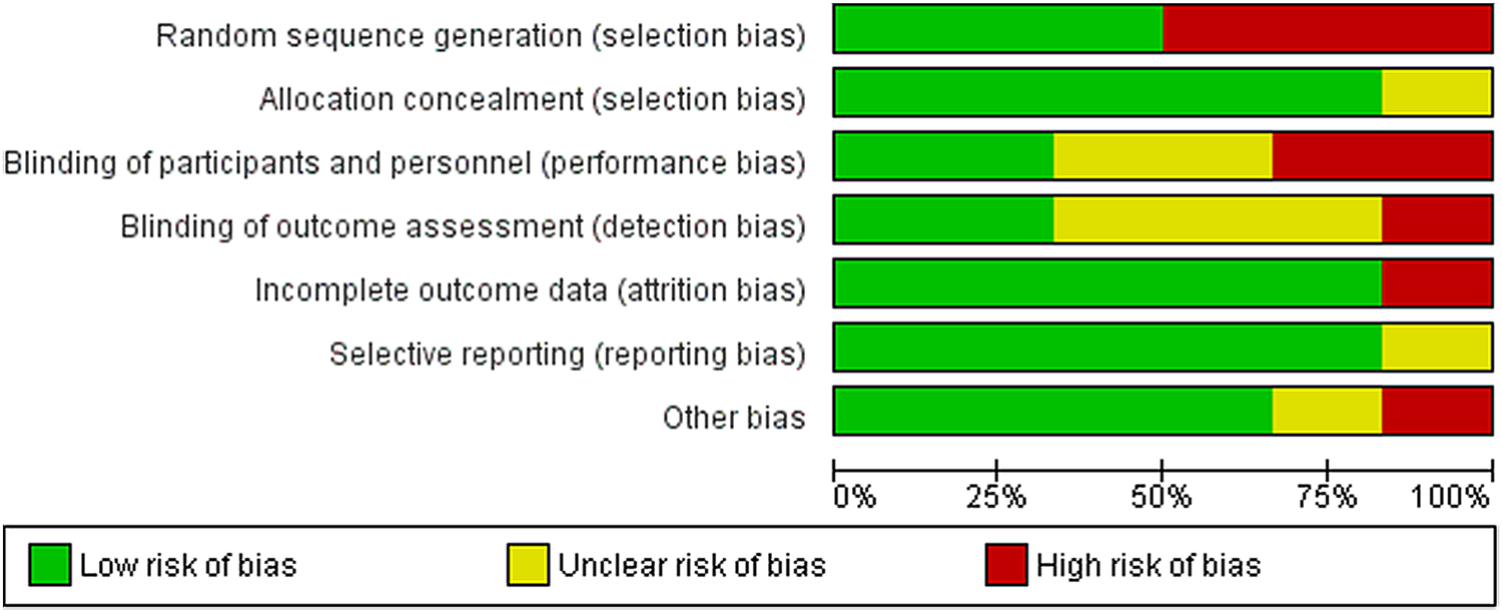
Methodological quality of the studies included in the meta-analysis

Continuous data were analysed using the Inverse Variance model and reported as mean difference. Forest plots were used for visualisation of the results. When the extracted data were appropriate for pooled analyses (e.g., similar techniques and patients), a meta-analysis was performed by a qualified statistician with specialised expertise in the field of meta-analyses. The included studies were evaluated for methodological flaws using the Cochrane Collaboration’s risk of bias assessment tool (Review Manager version 5.3). Seven domains of risk of bias were assessed for each study, including random sequence generation, allocation concealment, blinding of participants and personnel, blinding of outcome assessment, incomplete outcome data, selective reporting, and other bias. Figure 1 shows the overall risk of bias by domain: the risk of bias is displayed as low risk (green), unclear (yellow), or high risk (red) (Fig. 1). The heterogeneity of studies was calculated using the *I*^2^ index. An *I*^2^ value of 0–25% represents insignificant heterogeneity; > 25%–50% low heterogeneity; > 50%–75% moderate heterogeneity; and > 75% high heterogeneity [39]. The heterogeneity was considered by the random effects model. When different reporting pattern was detected, mean and standard deviation values were transformed according to Hozo et al. [40].

When several mean values and standard deviations were given in a study, these were weighted according to the number of patients. Analytical power was assessed through a posthoc power analysis of each meta-analysis.

In the study of Chen [31], the score for the AOFAS Group was not presented in the study. This mean value with the standard deviation was estimated by the other AOFAS studies in this meta-analysis (weighting according to the number of patients).

The Newcastle–Ottawa Scale was used for assessing the quality of observational studies. The SAS software, release 9.4 (SAS Institute Inc., Cary, NC, USA) was used to estimate and weight the mean values. For meta-analysis calculations, the Review Manager version 5.3 (The Cochrane Collaboration, The Nordic Cochrane Centre, Copenhagen, Denmark) was used. A *p* value of less than 0.05 was considered as statistically significant.

## Results

**Fig. 2:**
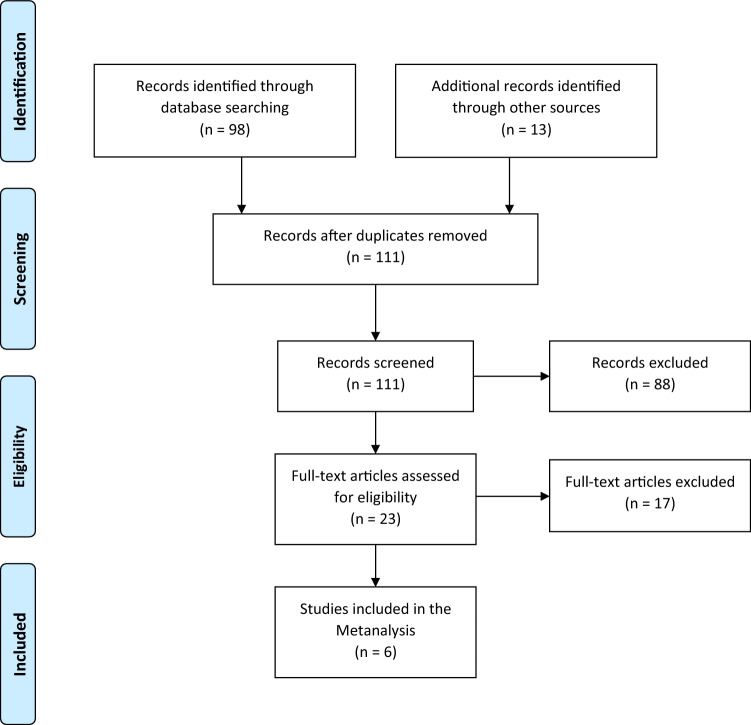
Study selection flow diagram

Applying the above-mentioned search strategy, 111 publications were identified and further analysed. After screening titles and abstracts/full texts, 92 publications were excluded. 19 studies (9 retrospective and 10 prospective) which described the incidence of CL and/or OCL were systematically reviewed. Six studies (retrospective patient identification and prospective follow-up) were eligible to be included in the meta-analysis (Fig. 2).

In 16 articles, cartilage damage was assessed through an arthroscopy of the joint, in two publications through MRI and in one study through inspection of the talus during the operation.

One study only included Wagstaffe fractures and one study only included Maisonneuve fractures. Two articles assessed only cartilage damage affecting the talus. Mean age of all included studies was 41.6 ± 8 years [95% CI 37.49–45.72]. The studies involved 1501 patients (44% females and 56% males).

**Fig. 3:**
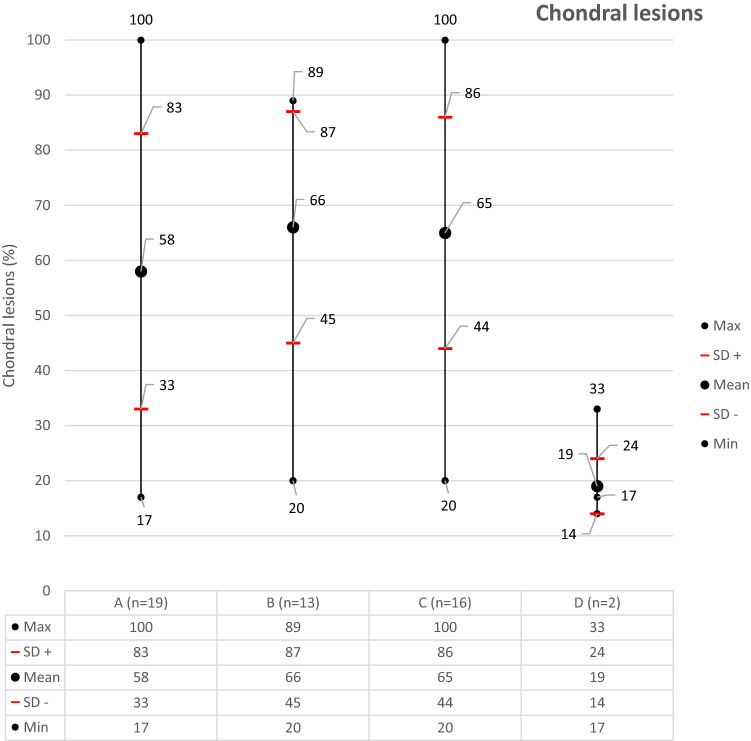
Incidence of CL and/or OCL according to number of studies included and diagnostic method. A: incidence of CL and/or OCL including all studies. B: incidence of CL and/or OCL including studies involving all types of ankle fractures and evaluating through arthroscopy the whole ankle (studies involving only Maisonneuve and Wagstaffe fractures or evaluating only the talus were excluded). C: incidence of CL and/or OCL including all studies using arthroscopy for evaluation. D: incidence of CL and/or OCL including all studies using MRI for evaluation. *n* number of studies, *SD* standard deviation

The mean incidence of CL and/or OCL in all mentioned studies was 58 ± 25% [95% CI 48.05–71.21]. The frequency of the CL and/or OCL ranged from 17% [12] to 89% [18]. To reach a more homogenous study selection, the studies including only Maisonneuve or Wagstaffe fractures and the studies assessing only the talus were excluded. The mean value of CL and/or OCL increased to 66 ± 21% [95% CI 44.88–70.37]. The mean incidence of CL and/or OCL using arthroscopy was 65 ± 21% [95% CI 53.9–76.72] and using MRI was 19% (Fig. 3). A detailed overview of the included publications is to be found in Table 1.

**Table 1:** Literature overview describing the incidence of concomitant chondral and/or osteochondral lesions after ankle fractures

	Author	Year	Study Design	*n*	Fracture Type^e,f^ *n* (%)	Assessment	Chondral lesions *n* with/without (%)
1	Chen et al. [31]	2019	Retrospective	36	23 (64) SER 13 (36) PER	ASK	26/36 (72)
2	Da Cunha et al. [32]	2018	Retrospective	116	87 (75) SER 27 (23) PER 1 (1) SA 1 (1) PA	ASK	90/116 (78)
3	Zhang et al. [34]^a^	2018	Retrospective	13	13 (100) SER	ASK	8/13 (62)
4	Fuchs et al. [23]	2016	Retrospective	42	26 (62) type B 16 (38) type C	ASK	26/42 (62)
5	Swart et al. [56]	2014	Prospective	12	6 (50) bimalleolar 6 (50) trimalleolar	ASK	5/12 (42)
6	Yan et al. [33]	2011	Retrospective	42	26 (62) SER 16 (38) PER	ASK	31/42 (74)
7	Stufkens et al. [15]	2010	Prospective	109	16 (15) type A 74 (78) type B 19 (17) type C	ASK	233/288 (81)
8	Leontaritis et al. [13]	2009	Retrospective	84	31 (37) PER 1 (1) SA 52 (62) SER	ASK	61/84 (73)
9	Boraiah et al. [12]	2009	Retrospective	153	128 (84) SER 10 (6) SA 12 (8) PER 3 (2) others	MRI	26/153 (17)
10	Aktas et al. [1]	2008	Retrospective	86	86 (100) SER	ASK	24/86 (28)
11	Yoshimura et al. [27]^b^	2008	Prospective	4	4 (100) Maisonneuve	ASK	4/4 (100)
12	Takao et al. [55]	2004	Prospective	41	13 (32) SER 28 (68) PA	ASK	30/41 (73)
13	Ono et al. [14]	2004	Prospective	105	58 (55) SER 15 (14,5) SA 15 (14,5) PA 17 (16) PER	ASK	21/105 (20)
14	Loren et al. [24]	2002	Prospective	48	24 (50) SER 10 (21) PER 5 (10) SA 4 (9) PA 5 (10) others	ASK	30/48 (63)
15	Thordarson et al. [18]	2001	Prospective	9	7 (78) SER 2 (22) PER	ASK	8/9 (89)
16	Hintermann et al. [17]	2000	Prospective	288	14 (5) type A 198 (69) type B 76 (26) type C	ASK	228/288 (79)
17	Sorrento et al. [3]^c^	2000	Retrospective	50	50 (100) SER	Ins-pection	19/50 (38)
18	Elsner et al. [42]	1996	Prospective	21	2 (10) type A 17 (80) type B 2 (10) type C	MRI	7/21 (33)
19	Lantz et al. [30]^d^	1991	Prospective	63	7 (11) type A 37 (59) type B 19 (30) type C	ASK	31/63 (49)

*n* number of patients, *ASK* arthroscopy, *MRI* magnetic resonance imaging

^a^Study including Wagstaffe Fractures

^b^Study including Maisonneuve Fractures

^c^Assessment of cartilage damage was performed by inspecting the Talus in the setting of the open reduction and internal fixation

^d^Study including assessment of cartilage damage on talus only

^e^Lauge-Hansen classification [62]: SER supination external rotation PER pronation external rotation SA supination adduction PA pronation abduction

^f^Danis–Weber classification [63]: type A, type B and type C

The cumulative meta-analysis sample size comprised a total of 400 patients (170 with and 230 without CL and/or OCL) available for a mean follow-up 23.9 ± 11.56 months [95% CI 11.79–36.07]. The average age of patients included in the meta-analyses was 44.3 ± 5.5 years [95% CI 38.57–50.13].

In five studies [1, 31–34] (*n* = 293), the assessment of cartilage damage was done through an arthroscopy during ORIF. Once detected, the cartilage damage was treated in all five publications using techniques such as debridement, chondroplasty or microfracture. In the publication of Zhang et al. [34], there was no detailed information provided regarding the treatment strategy of the detected CL and/or OCL.

In one MRI-based study [12] (*n* = 153), the preoperatively detected cartilage lesions were not addressed intraoperatively.

All included studies in the meta-analysis compared two groups clinically: with and without CL and/or OCL. The clinical outcome was evaluated in four studies (*n* = 177) using AOFAS and in two studies (*n* = 269) using FAOS.

**Fig. 4:**
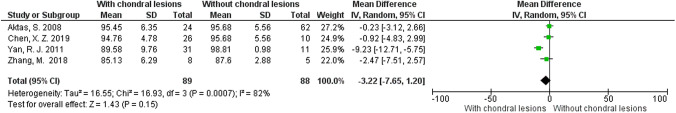
Meta-analysis using AOFAS to evaluate patients with CL and/or OCL after ankle fractures

The meta-analysis showed slightly higher mean AOFAS values in the group of patients without CL and/or OCL. However, the results were not statistically significant (*p* = 0.15) (Power 15%) (Fig. 4).

**Fig. 5:**
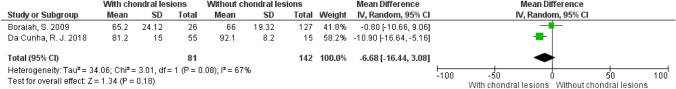
Meta-analysis using FAOS to evaluate patients with CL and/or OCL after ankle fractures

Regarding the studies that used FAOS, the mean values were moderately higher in the group of patients without CL and/or OCL (*p* = 0.18) (Power 100%) (Fig. 5). The highest discrepancy in clinical outcomes between the defined group was documented by Da Cunha et al. [32] with an mean FAOS difference of 10.9 points and Yan et al. [33] with a mean AOFAS difference of 9.23 points, both in favour of the group of patients without CL and/or OCL.

A detailed overview of the included publications in the meta-analyses is to be found in Table 2.

**Table 2:** Included studies in the meta-analyses

Author	Year of publication	Study design	Age in years mean ± SD (95% CI)	Number of patients (n)	Gender (F/M)	Follow-up months mean ± SD (95% CI)	Patients available for follow-up (*n*)	Cartilage assessment	Score	Clinical Outcome					
										With CL/OCL			Without CL/OCL		
										Mean	SD	*n*	Mean	SD	*n*
Chen et al. [31]	2019	Retrospective identification, prospective follow-up	47	36	22/14	41.7	36	Arthroscopy	AOFAS	94.76	4.78	26	95.68	5.56	10
Da Cunha et al. [32]	2018	Retrospective identification, prospective follow-up	42.7	116	50/66	20.8	70	Arthroscopy	FAOS	81.2	15	55	92.1	8.2	15
Zhang et al. [34]	2018	Retrospective identification, prospective follow-up	47.2	13	4/9	14.3	13	Arthroscopy	AOFAS	85.13	6.29	8	87.6	2.88	5
Boraiah et al. [12]	2009	Retrospective identification, prospective follow-up	51.8	153	89/64	20.9	153	MRI	FAOS	65.20	24.12	26	66	19.32	127
Aktas et al. [1]	2008	Retrospective identification, prospective follow-up	41.4	86	38/48	33.9	86	Arthroscopy	AOFAS	95.45	6.35	24	95.68	5.56	62
Yan et al. [33]	2011	Retrospective identification, prospective follow-up	36	42	17/25	12	42	Arthroscopy	AOFAS	89.58	9.76	31	98.81	0.98	11
			44.3 ± 5.5 [95% CI 38.57–50.13]	446		24 ± 11.5 [95% CI 11.79–36.07]	400					170			230

*SD* standard deviation, *n* number of patients, *Gender F/M* female/male, *CI* confidence interval

## Discussion

### Incidence of CL and/or OCL

The mean incidence of CL and/or OCL in all included studies (*n* = 19) involved heterogenic ankle fracture morphologies, regardless of the method used to assess the cartilage damage was 58 ± 25% [95% CI 48.05–71.21]. The mean incidence of CL and/or OCL calculated including comparable studies (*n* = 13) evaluating arthroscopically the whole joint and including all types of fractures was 66 ± 21% [95% CI 44.88–70.37]. Similar results were described by Zhang et al. [34], Fuchs et al. [23] 62% and Loren et al. [24] with 63%.

The publications describing the incidence of CL and/or OCL evaluated through MRI (mean value 19%) showed wide inconsistency as compared to arthroscopic diagnostics. Yasui et al. [41] found that the estimation of osteochondral lesions of the talus between MRI and arthroscopy was inconsistent, due to geometry of the articular surface that limits the exact measurement of the lesions in MRI, since axial cuts of the convex talus restrict the precise visualisation of the lesions. Furthermore, the mobility of the joint might restrict the arthroscopic accessibility of all compartments. However, unlike the results of the present systematic review including only few MRI-based studies, Yasui et al. [41] showed an overestimation of the size of lesions in MRI in 53.3% of the cases (*p* = 0.03). Even though all authors [12, 41, 42] performed comparable imaging techniques including similar repetition time/echo time, T1/T2 imaging and 3 mm slice thickness. A possible explanation may be the time difference between the publications (Yasui et al. [41], Elsner et al. [42] and Boraiah et al. [12]) and the improved MRI qualities. Another factor that may have played a role is the different time interval between the MRI assessment of the traumatic chondral lesions and their assessment in arthroscopy. In the publication of Yasui et al. [41], the time interval between MRI and arthroscopy was 37.3 ± 10.1 days. In the publications listed in Table 1, the MRI or arthroscopic assessment of chondral lesions was performed shortly after trauma (4.4 ± 1.5 days).

The methods used to diagnose CL and/or OCL include MRI [43, 44], CT [45] and arthroscopy [1, 46–48]. CT scans have the disadvantage of not being able to visualise isolated chondral lesions [45]. Osteochondral injuries were able to be identified with CT scans [45]. Van Bergen et al. [49] reported a 0.81 sensitivity, 0.99 specificity, 0.96 positive predictive value (PPV) and 0.94 negative predictive value (NPV) of CT scanning in the diagnosis of talar osteochondral defects, against, respectively, 0.96, 0.96, 0.89 and 0.99 using MRI. As a result, many authors used MRI [43, 44] to identify reliably chondral, osteochondral and subchondral lesions; however, MRI tends to overestimate their extent [9, 29]. According to Matilla et al. [50], the sensitivity of MRI in detecting grade I–II chondral damage was 66% and up to 100% when it comes to diagnosing grad III–IV lesions as graded by Shahriaree et al. [51]. Nakasa et al. [52] reported a sensitivity of 71.2% and a specificity of 71.3% of MRI in the diagnosis of cartilage lesions. In spite of the continuously evolving techniques and the high-resolution imaging, the standard 3 mm slice thickness of MRI may also miss some chondral lesions since the average thickness of talus cartilage, according to the publication of Sugimoto et al. [53], is thinner and varies between 1.35 ± 0.22 mm in males and 1.11 ± 0.28 mm in females.

Consequently, many authors consider arthroscopy to be the most reliable diagnostic method to assess cartilage lesions since it allows the surgeon to evaluate for CL and/or OCL and their extent under direct vision [1, 46–48]. Limitations of the diagnostic arthroscopy of the ankle joint are inability to detect subchondral lesions and the compromised accessibility of all joint compartments, especially the posterior portion with ventral portals [31]. In a way to address the limited visualisation of the talar dome, many authors recommend performing the arthroscopy in plantarflexion. Hirtler et al. [54] proved in a cadaver study the superiority of maximal plantarflexion over non-invasive distraction in anterior ankle arthroscopy. Another limitation of arthroscopy in the setting of acute fracture is the swelling of periarticular soft tissues caused by fluid pressure during arthroscopy. This may lead to a more difficult visualisation of the joint and to a compromised wound closure and healing. For this reason, some authors perform the arthroscopy using CO_2_ instead of saline and a tourniquet to optimise the visibility in the joint [17]. In studies included in the present meta-analysis, the arthroscopy was performed using two portals: anteromedial and anterolateral [14, 27, 31, 32, 55] or one anteromedial portal and the anterolateral portal only when necessary [17, 23, 56]. Loren et al. used an additional third posterolateral portal [24] and detected CL and/or OCL in 63% of the patients with acute ankle fractures. Their findings concerning the incidence of CL and/or OCL are in line with the results of the present systematic review. This may be due to the fact that the additional posterolateral portal helped diagnosing posterior lesions that could be missed in the standard anterior-portal arthroscopy. In fact, several publications [28, 57] showed that the posterolateral compartment frequently revealed chondral lesions. For this reason, posterior portals are gaining more attention despite the challenging technique and possible complications. In addition, to improve the accessibility of the posterior talus even more, some authors [54, 58] suggest performing the arthroscopy under traction. Barg et al. [58] showed in a cadaver study the significant positive effect of axial traction in posterior arthroscopy on the visualisation of all segments of the talus both non-invasively with strapping and using a wire distractor through the calcaneus.

### Clinical outcome with and without CL and/or OCL

The performed meta-analysis revealed that CL and/or OCL after ankle fractures have a negative effect on the clinical outcome, as measured with FAOS and AOFAS, however without reaching statistical significance.

Chen et al. [22], Zhang et al. [34] and Aktas et al. [1] used AOFAS for evaluation and did not find any difference between the patients with and those without CL and/or OCL. Yan et al. [33] used the same score and reported significantly better clinical outcome without CL and/or OCL. The latter may be due to the short follow-up period of 12 months. Due to the high heterogeneity of the data using AOFAS, the clinical significance should be carefully regarded. In the long-term follow-up of 12.9 years, Stufkens et al. [15] found that the presence of CL and/or OCL had a statistically significant effect on the development of clinical and radiographic osteoarthritis. Patients with cartilage lesions had a 3.5-fold higher chance of developing radiographic osteoarthritis (Kannus arthritis score < 90) and a 5-fold higher risk of having an unsatisfying long-term clinical outcome (AOFAS score < 90). As for the localisation, anterior and lateral talar lesions as well as medial malleolar lesions were found to significantly raise the possibility of developing posttraumatic osteoarthritis.

The results of the present meta-analysis using FAOS also revealed no statistically significant difference between the defined groups. The data heterogeneity of the meta-analysis with FAOS was moderate. Da Cunha et al. [32] and Boraiah et al. [12] used the same FAOS score comparing patients with CL and/or OCL after ankle fractures but the reported results showed high discrepancy (Mean score difference between groups 10.9 vs 0.8 points). The variability of the results between both studies [12, 32] might be due to the different diagnostic method utilised (arthroscopy vs MRI). MRI seems to overestimate the size/depth of CL and/or OCL [59]. However, there are limited data available in the literature comparing MRI and arthroscopy in the same population regarding diagnostic accuracy to detect CL and/or OCL. Boraiah et al. [12] addressed the detected cartilage damage arthroscopically, which might have had a positive effect on the clinical outcome. Also, high-quality data are lacking comparing randomly the natural course with several treatment methods of CL and/or OCL.

Similarly, various authors [14, 18, 23, 25, 29, 30] analysed the clinical outcome after CL and/or OCL but used different outcome measures. Therefore, they could not be included in the meta-analyses. Lantz et al. [30], Lorez et al. [25] and Fuchs et al. [23] found a poorer clinical outcome in patients with cartilage damage after ankle fractures using a 100-point scoring system, Kitaoka score and the Olerud/Molander scoring system, respectively. Regier et al. [29] reported similar results using AOFAS but did not report any mean values of the score for each group.

Thordarson et al. [18] found no significant clinical effect of posttraumatic cartilage damage using MODEMs score and SF 36. Ono et al. [14] used the method of Burwell and Charnley to assess clinical outcome (good, fair or poor). The clinical outcome was good for all patients without significant differences regardless of cartilage damage.

One of the limitations of the available data was the diversity of scores used to assess clinical outcomes. In addition, some follow-up periods were inhomogeneous. However, the mean follow-up of all included studies with both scores was 24 months. Several studies that measured the outcome using other scores had to be excluded from the meta-analysis. Though, this exclusion increased the homogeneity of the used scores and improved the quality of the meta-analysis. Another limitation is the validity of one of the used scores, namely the AOFAS, which is only partially validated (only structural and criterion validity), thus weakening its informative value and significance [60].

Furthermore, some of the publications included in this meta-analysis did not perform any CT or MRI postoperatively in context of the follow-up and restricted their monitoring to native radiographs. Most of the fractures with concomitant chondral lesions resulted from severely dislocated fractures [12, 28, 31, 32] and were associated with syndesmotic disruption and ligamentous injury [31, 32]. Reconstruction and healing of these anatomic structures are being considered as cofounders and also determine the clinical outcome [61].

Another limitation is the small sample size of the studies included, even though the total number of patients included in this meta-analysis is adequate. This limitation was counteracted by the fact that the mean values and standard deviations given in each study were weighted according to the number of patients. Based on these results, the long-term effect of the CL and/or OCL remains unclear.

## Conclusions

The incidence of CL and/or OCL after ankle fractures is high. However, there is a certain variability of the incidence of CL and/or OCL depending on the fracture type and diagnostic modality.

The clinical outcome, as assessed with AOFAS/FAOS, revealed similar values regardless of the presence of CL and/or OCL after ankle fractures.

Studies with higher sample size and homogeneous assessment methods are warranted to evaluate the short-, mid- and longer-term clinical outcomes after ankle fractures with CL and/or OCL.

## Abbreviations

AOFAS: American Orthopaedics Foot and Ankle Society ankle–hindfoot scale
CCCL: Concomitant chondral lesions
CI: Confidence interval
COCOCL: Concomitant osteochondral lesions
CT: Computer tomography
FAOS: Foot and Ankle Outcome Scoring
MODEMS: Musculoskeletal outcomes data evaluation and management scale
MRI: Magnetic resonance imaging
ORIF: Open reduction and internal fixation
PA: Pronation abduction
PER: Pronation external rotation
PRISMA: Preferred reporting items for systematic reviews and meta-analyses
SA: Supination adduction
SER: Supination external rotation
SF-36: Short Form Health 36
