# Correction to: Incidence of concomitant chondral/osteochondral lesions in acute ankle fractures and their effect on clinical outcome: a systematic review and meta-analysis

**DOI:** 10.1007/s00402-021-03985-y

**Published:** 2021-06-22

**Authors:** Ali Darwich, Julia Adam, Franz-Joseph Dally, Svetlana Hetjens, Ahmed Jawhar

**Affiliations:** 1 Department of Orthopaedics and Traumatology Surgery, University Medical Centre, Medical Faculty Mannheim of the University of Heidelberg, Theodor-Kutzer-Ufer 1-3, 68167 Mannheim, Germany; 2 Institute of Medical Statistics and Biomathematics, University Medical Centre, Medical Faculty Mannheim of the University of Heidelberg, Mannheim, Germany; 3 Department of Trauma, Hand and Reconstructive Surgery, Klinikum Worms, Academic Teaching Hospital of the University Mainz, Worms, Germany

## Correction to: Archives of Orthopaedic and Trauma Surgery (2021) 141:63–74 10.1007/s00402-020-03647-5

The article Incidence of concomitant chondral/osteochondral lesions in acute ankle fractures and their effect on clinical outcome: a systematic review and meta‑analysis, written by Ali Darwich, Julia Adam, Franz‑Joseph Dally, Svetlana Hetjens and Ahmed Jawhar, was originally published Online First without Open Access. After publication in volume 141, issue 1, page 63–74 the author decided to opt for Open Choice and to make the article an Open Access publication. Therefore, the copyright of the article has been changed to © The Author(s) 2021 and this article is licensed under a Creative Commons Attribution 4.0 International License, which permits use, sharing, adaptation, distribution and reproduction in any medium or format, as long as you give appropriate credit to the original author(s) and the source, provide a link to the Creative Commons licence, and indicate if changes were made. The images or other third party material in this article are included in the article’s Creative Commons licence, unless indicated otherwise in a credit line to the material. If material is not included in the article’s Creative Commons licence and your intended use is not permitted by statutory regulation or exceeds the permitted use, you will need to obtain permission directly from the copyright holder. To view a copy of this licence, visit http://creativecommons.org/licenses/by/4.0/.

The original article has been corrected.

